# Veliparib in combination with whole-brain radiation therapy for patients with brain metastases from non-small cell lung cancer: results of a randomized, global, placebo-controlled study

**DOI:** 10.1007/s11060-016-2275-x

**Published:** 2016-09-21

**Authors:** Pierre Chabot, Te-Chun Hsia, Jeong-Seon Ryu, Vera Gorbunova, Cristobal Belda-Iniesta, David Ball, Ebenezer Kio, Minesh Mehta, Katherine Papp, Qin Qin, Jane Qian, Kyle D. Holen, Vince Giranda, John H. Suh

**Affiliations:** 1 Hôpital Maisonneuve-Rosemont, Montreal, QC Canada; 2 China Medical University Hospital, China Medical University, Taichung, Taiwan People’s Republic of China; 3 Inha University Hospital, Jung-Gu, Incheon, South Korea; 4 N.N. Blokhin Russian Cancer Research Center, Moscow, Russia; 5 Centro Integral Oncológico Clara Campal HM Sanchinarro, Madrid, Spain; 6 Peter MacCallum Cancer Centre, Melbourne, VIC Australia; 7 IU Health Goshen Center for Cancer Care, Goshen, IN USA; 8 University of Maryland, College Park, MD USA; 9 AbbVie Inc., Chicago, IL USA; 10 Cleveland Clinic, 9500 Euclid Ave, Cleveland, OH 44195 USA

**Keywords:** Veliparib, PARP inhibitor, Whole-brain radiation therapy, Brain metastases, Non-small cell lung cancer, Randomized clinical trial

## Abstract

Veliparib is a potent, orally bioavailable, poly (adenosine diphosphate-ribose) polymerase (PARP) inhibitor that crosses the blood–brain barrier and has been shown to potentiate the effects of radiation in preclinical and early clinical studies. This phase 2, randomized, global study evaluated the efficacy and safety of veliparib in combination with whole-brain radiation therapy (WBRT) in patients with brain metastases from non-small cell lung cancer (NSCLC). Three-hundred and seven patients with brain metastases from NSCLC were randomized 1:1:1 to WBRT (30 Gy in 10 fractions) plus 50 mg veliparib twice daily (BID; *n* = 103), 200 mg veliparib BID (*n* = 102), or placebo BID (*n* = 102). Treatment began within 28 days of diagnosis. Tumor response and safety were assessed; the primary endpoint was overall survival (OS). Patients who received ≥1 dose of treatment were included in the safety analysis. All randomized patients were included in the efficacy endpoint analyses. Patient characteristics were well balanced between treatment arms. Median OS was 185 days for patients treated with WBRT plus placebo and 209 days for WBRT plus veliparib (50 or 200 mg). No statistically significant differences in OS, intracranial response rate, and time to clinical or radiographic progression between any of the treatment arms were noted. No differences were observed in adverse events (all grades) across treatment arms; nausea, fatigue, alopecia, and headache were the most commonly reported. No new safety signals were identified for veliparib. A significant unmet need for therapies that improve the outcomes of patients with brain metastases from NSCLC remains.

**Electronic supplementary material:**

The online version of this article (doi:10.1007/s11060-016-2275-x) contains supplementary material, which is available to authorized users.

## Introduction

Brain metastases are the most common intracranial malignancy affecting up to 30 % of patients diagnosed with cancer with an estimated annual incidence of more than 200,000 cases in the USA [1]. Approximately 7.4 % of patients with non-small cell lung cancer (NSCLC) will present with brain metastases at diagnosis; an additional 25–30 % will develop brain metastases over the course of their disease [2]. These patients have a very poor prognosis (median survival between 3 and 4.9 months) and an impaired quality of life (QoL) with significant neurologic, cognitive, and emotional impairment [3].

Whole-brain radiation therapy (WBRT) is the standard of care for patients with brain metastases that are surgically unresectable or have multiple lesions [1]. Treatment is associated with improvement in neurologic symptoms and decreased neurologic death [4]. In candidates suitable for resection or radiosurgery, the therapeutic value of WBRT is supported by level 1 evidence in terms of further reduction in intracranial failure [5–7]. However, treatment is likely associated with cognitive decline and the impact on survival remains controversial [8–10]. As a result, the optimal management of patients with brain metastases is controversial, given the array of treatment options and strong opinions regarding the utilization of these options [11].

The combination of WBRT and agents with radiosensitizing properties has been evaluated to improve upon the results of WBRT alone [12, 13]. Randomized, controlled trials have failed to demonstrate a categorical benefit in this setting for either local brain tumor control or overall survival (OS) [14, 15]. Therefore, there remains a significant unmet medical need for patients with multiple brain metastases.

Poly (adenosine diphosphate-ribose) polymerase (PARP) is a family of enzymes involved in a number of cellular processes, including DNA replication, transcription, and cell death [16]. Increased PARP activity has been observed in numerous cancers, and is thought to be one possible mechanism of resistance to cell-death by DNA-damaging therapeutics. There is evidence that the absence of PARP-1 and -2, which are both activated by DNA damage and facilitate DNA repair, results in hypersensitivity to ionizing radiation [17]. Therefore, the inhibition of PARP-mediated DNA damage repair can help sensitize cells to DNA-damaging agents.

Veliparib (ABT-888) is a potent, orally bioavailable, PARP-1 and -2 inhibitor that has the ability to cross the blood–brain barrier. In preclinical models, veliparib potentiated the antitumor activity of fractionated radiation [18, 19] and inhibited PARP levels in patient tumors in a phase 0 biopsy trial at doses as low as 25 mg [20]. A phase 1 study of veliparib in combination with WBRT was then initiated to understand the safety and tolerability in patients with brain metastases. The study included patients with brain metastases from a variety of tumor types, and escalated the dose of veliparib from 10 to 300 mg. Veliparib was associated with infrequent Grade 3 toxicities up to doses as high as 300 mg twice daily (BID); however, 200 mg BID was selected as the recommended phase 2 dose due to frequent Grade 2 nausea events. In a cohort of 15 patients with brain metastases from NSCLC, tumor responses (46 % versus 25–30 %) and median survival (10.5 months versus 3–4.5 months) were increased compared with historical data [21–24]. Thus, both the preclinical and early clinical studies supported the randomized phase 2 evaluation of combination therapy with veliparib and WBRT in patients with brain metastases from NSCLC. Two doses of veliparib were evaluated (50 and 200 mg) based on the expected efficacious dose from the phase 0 study and the recommended phase 2 dose from the phase 1 study, respectively. The primary endpoint for the trial was OS. Given that OS is a difficult endpoint in this population due to the effects of non-cranial disease, additional endpoints were measured to look for potentiation of radiation on the local, intracranial disease. These endpoints included radiographic tumor progression, clinical progression, QoL, and neurocognitive abilities.

## Methods

This global phase 2, randomized, double blinded, multicenter study evaluated WBRT in combination with veliparib or placebo in patients with brain metastases from NSCLC. The study consisted of a treatment period, and a long-term follow-up period. During the long-term follow-up period, study visits for safety, radiographic response and neurologic assessments were performed monthly for 9 up to 24 months, and survival and post-treatment therapy data were collected at 2-monthly intervals for 6 months beginning at month 2 and every 3 months afterwards for up to 36 months, or as needed for more frequent data collection. The study objectives were to evaluate the efficacy and safety of WBRT administered in combination with veliparib BID (50 or 200 mg) versus placebo BID. The primary endpoint was OS. Secondary endpoints included best tumor response, time to intracranial radiographic progression, and time to clinical brain metastasis progression.

Eligible patients had cytologically or histologically confirmed NSCLC and brain metastases demonstrated via magnetic resonance imaging (MRI) brain scan. Total number of brain metastases was not a part of inclusion criteria. Patients had to be over the age of 18 years and be eligible for WBRT treatment (per investigator), with Karnofsky performance status (KPS) scores ≥70, and have adequate hematologic, renal, and hepatic function. Patients could not have been diagnosed with brain metastases >28 days before commencing treatment or have received prior cranial radiation or undergone resection for brain metastases. To exclude patients who might be more likely to die from systemic disease as opposed to neurologic disease, additional exclusion criteria included more than two sites of metastases from NSCLC (excluding the brain, bone, and thorax) and evidence of liver metastases. Due to the very poor outcomes for patients with leptomeningeal metastases and subarachnoid spread of the tumor, these patients were excluded.

**Fig. 1:**
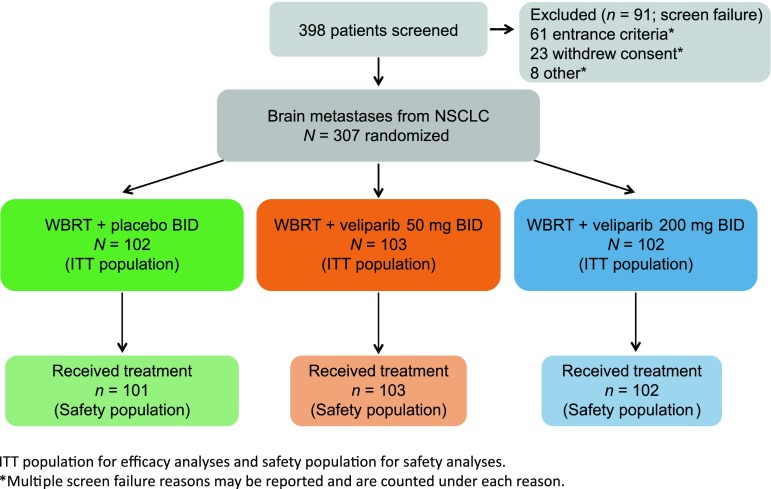
Study design. *BID* twice daily, *ITT* intent to treat, *NSCLC* non-small cell lung cancer, *WBRT* whole-brain radiation therapy

Baseline neurologic assessment, QoL evaluations, and activities of daily living assessments were performed ≤7 days before randomization. Patients were randomized 1:1:1 to WBRT plus veliparib BID (50 or 200 mg) or placebo BID (Fig. 1). Patients were stratified by graded prognostic assessment (GPA) score (≤2.5 versus >2.5) and neurologic symptoms (symptomatic versus asymptomatic). The treatment period began on the first day of WBRT and continued for 45 days. The follow-up period then consisted of patient visits that included QoL measurements, assessment of vital signs and KPS scales, clinical laboratory test, physical and neurologic examinations, and MRIs which continued for 3 years or until time of death, whichever was first. Treatment was required to begin within 28 days of the diagnosis of brain metastases. All patients received 30.0 Gy of WBRT in ten daily fractions of 3.0 Gy, given 5 days per week (excluding holidays and weekends). Oral veliparib BID (50 or 200 mg) or placebo BID was self-administered starting on day 1 of WBRT and continued until 1 day after completion of WBRT, including weekends and holidays. Study visits and procedures during the treatment period were performed on treatment days 1, 8, and the last day of treatment with WBRT. During the treatment period, if a patient discontinued veliparib/placebo and WBRT due to both radiographic and clinical brain metastases progression, the patient continued to be followed for survival and post-treatment therapy data for up to 36 months.

During the follow-up period, study visits and safety assessments (physical examinations, vital signs, KPS, and laboratory tests), radiographic assessments (brain MRIs) and neurologic assessments (standardized clinical neurologic examination, physician inventory of neurologic symptoms, and neurocognitive test battery) were performed monthly (30-day intervals) for 9 months, and every 3 months thereafter for up to 24 months.

Due to the potential of long-term sequelae from veliparib combined with WBRT, after 3 years of follow-up, patients who had not previously had clinical progression of disease were offered continued neurologic assessments until they experienced clinical brain metastases progression (with or without radiographic progression), or discontinued for any other reason (including death). During this time, study visits were performed every 3 months. Radiographic brain metastases progression was assessed by the investigator at each study site and independently by a central imaging center. Radiographic response or progression was modeled after the Macdonald criteria [25] with response evaluation criteria in solid tumors (RECIST) definitions of measurable lesions and non-target lesions [26, 27].

The distribution of the primary endpoint OS was estimated for each treatment group using Kaplan–Meier methodology and compared between WBRT plus veliparib 50 mg BID and WBRT plus placebo BID, as well as between WBRT plus veliparib 200 mg BID and WBRT plus placebo BID treatment groups using the log-rank test stratified by GPA score (≤2.5 versus >2.5). All other time-to-event endpoints (time to clinical brain metastases progression, time to intracranial radiographic progression) were also analyzed using the same method as that for OS. The best tumor response rates were compared between WBRT plus veliparib 50 mg BID and WBRT plus placebo BID, as well as between WBRT plus veliparib 200 mg BID and WBRT plus placebo BID treatment groups using the Cochran–Mantel–Haenszel test stratified by GPA score (≤2.5 versus >2.5). Additional analyses were performed using a Cox proportional hazards model to explore the effect of baseline factors, including GPA score, neurologic symptoms, sex, age, region, and others.

Safety evaluations included the assessment of treatment-emergent adverse events (AEs) (e.g. those that had an onset on or after the first day of the first dose of study drug) throughout the study using the National Cancer Institute Common Terminology Criteria for Adverse Events (version 4.0). AEs were reported up to 30 days after completion of veliparib and placebo. After 30 days, investigators were requested to report any significant AEs (Grade 3/4) and/or serious AEs considered to be related to treatment. Patients who received ≥1 dose were included in the safety analyses. Fisher’s exact test was performed to compare the percentages of patients experiencing an AE between WBRT plus placebo BID versus WBRT plus veliparib BID (50 or 200 mg).

## Results

### Baseline characteristics

In total, 307 patients were randomly assigned to WBRT plus placebo BID (*n* = 102), WBRT plus 50 mg veliparib BID (*n* = 103) and WBRT plus 200 mg veliparib BID (*n* = 102). There was only one patient who was lost to follow-up for survival information, see the CONSORT diagram (Fig. 1). Baseline characteristics of the patients were generally well balanced among the treatment groups (Table 1). The median age (and range) of the treatment groups was 60 (41–86), 60 (33–83), and 62 (39–81) years, and 55, 59, and 65 % of patients were males, respectively. The most common subtype of NSCLC at diagnosis was adenocarcinoma (WBRT plus placebo BID, 80 %; WBRT plus 50 mg veliparib BID, 83 % and WBRT plus 200 mg veliparib BID, 75 %). The majority of patients had GPA scores ≤2.5 (89, 88, and 90 %, respectively) and KPS > 80 (61, 66, and 62 %, respectively).

**Table 1:** Patient demographics and baseline characteristics

	Placebo + WBRT (*N* = 102)	Veliparib 50 mg + WBRT (*N* = 103)	Veliparib 200 mg + WBRT (*N* = 102)
Age, median (range)	60 (41–86)	60 (33–83)	62 (39–81)
Sex, n (%)			
Male	56 (55)	61 (59)	66 (65)
Female	46 (45)	42 (41)	36 (35)
Race, n (%)			
White	79 (78)	85 (83)	66 (65)
African American	0 (0)	2 (2)	6 (6)
Asian	22 (22)	16 (16)	28 (28)
Region, n (%)^a^			
USA	15 (15)	12 (12)	9 (9)
Non-USA	87 (85)	91 (88)	93(91)
Histology at diagnosis, n (%)			
Adenocarcinoma	81 (80)	86 (83)	77 (75)
Squamous cell carcinoma	11 (11)	13 (13)	17 (17)
Large cell carcinoma	7 (7)	0 (0)	5 (5)
Other	2 (2)	4 (4)	3 (3)
Missing	1	0	0
GPA score, n (%)			
≤2.5	91(89)	91 (88)	92 (90)
>2.5	11 (11)	12 (12)	10 (10)
Neurological symptoms (IVRS/IWR), n (%)			
Asymptomatic	53 (52)	53 (52)	53 (52)
Symptomatic	59 (58)	50 (49)	49 (48)
KPS, n (%)			
≤80	39 (39)	35 (34)	39 (38)
>80	62 (61)	68 (66)	63 (62)
Missing	1	0	0
Number of brain mets (per central review), n (%)			
1	18 (18)	22 (22)	14 (14)
2–3	22 (22)	26 (26)	29 (19)
>3	58 (59)	53 (51)	56 (57)
Unknown/missing	4	2	3
Extracranial mets, n (%)			
Yes	68 (67)	76 (74)	74 (73)
No	33 (32)	27 (26)	28 (27)
Unknown	1	0	0
Smoking, n (%)^b^			
Current	25 (25)	22 (21)	20 (20)
Former	52 (51)	63 (61)	58 (57)
Never	24 (47)	18 (17)	23 (23)
EGFR, n (%)^c^			
No	34 (64)	35 (71)	35 (66)
Yes	19 (36)	14 (29)	18 (34)
Unknown	48	53	49
Missing	1	1	0
ALK, n (%)^c^			
No	26 (100)	24 (96)	27 (96)
Yes	0 (0)	1 (4)	1 (4)
Unknown	75	77	74
Missing	1	1	0

Percentages based on known data

*ALK* anaplastic lymphoma kinase, *EGFR* epidermal growth factor receptor, *GPA* graded prognostic assessment, *IVRS*/*IWR*, interactive voice/web response system, *KPS* Karnofsky performance status, *mets* metastases, *WBRT* whole-brain radiation therapy

^a^One patient in placebo arm is Native Hawaiian or Pacific islander

^b^Missing smoking status for one patient in placebo arm

^c^EGFR and ALK status was not required and the majority of sites did not report; percentages based on known data

### Study drug exposure

Study drug and WBRT exposure was similar across treatment arms; no statistical differences were observed between arms and all patients received the recommended study dose (Supplemental Table 1). Pharmacokinetic results confirmed drug levels for veliparib and no measurable drug exposures for those on placebo (data not shown).

### Efficacy

**Fig. 2:**
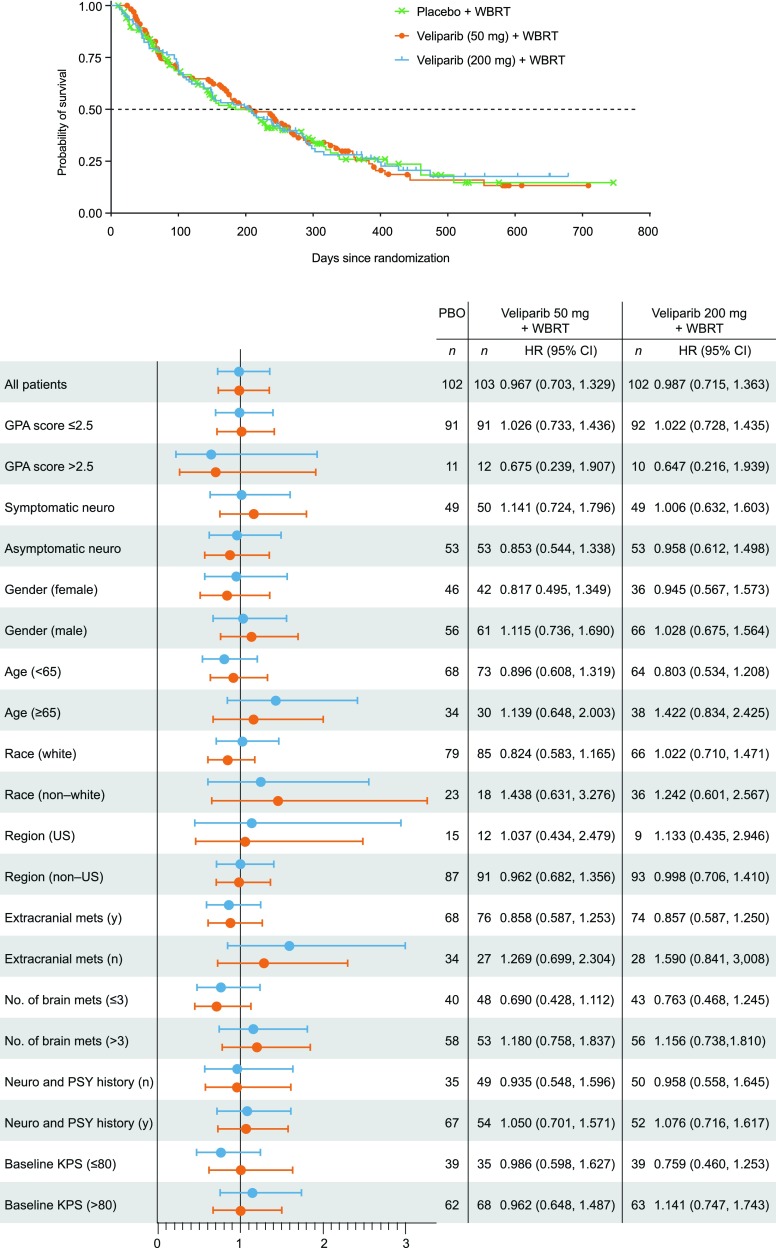
Overall survival—all randomized patients and subgroup analysis. *GPA* graded prognostic assessment, *KPS* Karnofsky performance score, *mets*, metastases, *neruo* neurologic, *PSY* psychologic, *WBRT* whole-brain radiation therapy

The median OS was 185 days for patients treated with WBRT plus placebo, 209 days for WBRT plus 50 mg veliparib (*p* = 0.927 versus placebo), and 209 days for WBRT plus 200 mg veliparib (*p* = 0.905 versus placebo; Table 2). There was no significant difference in OS between either of the WBRT plus veliparib (50 or 200 mg) arms and the WBRT plus placebo arm (Fig. 2).

**Table 2:** Summary of primary and secondary endpoints

	Placebo + WBRT (*N* = 102)^a^	Veliparib 50 mg + WBRT (*N* = 103)		Veliparib 200 mg + WBRT (*N* = 102)	
Median overall survival, days (95 % CI)	185 (137, 251)	209 (169, 264)	*p* = 0.933	209 (138, 255)	*p* = 0.909
Objective response rate, (%)	41.2	36.9	*p* = 0.535	42.2	*p* = 0.898
Median time to clinical brain metastasis progression^a^, days (95 % CI)	348 (216, NR)	286 (192, NR)	*p* = 0.864	255 (204, 342)	*p* = 0.301
Median time to radiographic brain metastasis progressionb, days (95 % CI)	259 (184, NR)	226 (147, 360)	*p* = 0.314	224 (137, 358)	*p* = 0.536

*p* values (against placebo)

*CI* confidence interval, *NR* not reached, *WBRT* whole-brain radiation therapy

^a^Per event review board

^b^Per central imaging center

OS was also evaluated for the following subgroups: GPA score, sex, age, geographic region, presence of extracranial metastases, number of brain metastases, baseline KPS, smoking status, asymptomatic/symptomatic neurologic impairment, neurologic and psychiatric history, and race. No significant difference in OS was detected amongst the different subgroups analyzed (Fig. 2). Investigator assessment of epidermal growth factor receptor (EGFR) mutations and anaplastic lymphoma kinase (ALK) rearrangements were collected; however, they were infrequently reported. Therefore, it was not possible to draw conclusions with the very limited numbers.

Evaluation of secondary endpoints (best tumor response rate, time to clinical brain metastases progression, and time to intracranial radiographic progression), also did not identify any significant differences between either of the veliparib (50 mg versus 200 mg) plus WBRT arms and the placebo plus WBRT arm (Table 2). Eighty-seven patients (33, 29, and 25) had radiographic progression found in either target lesions or new lesions; 41 (18, 12, and 11) had new lesions and 58 (18, 21, and 19) had progression of target lesions. The best tumor response rate (complete and partial responses) was also similar between the arms, at 41.2, 36.9, and 42.2 %. See Table 2 for full efficacy results. Additionally, there was no difference in change from baseline in neurocognitive tests measured by z-score across all scheduled visits between either veliparib dose groups (50 mg versus 200 mg) and placebo group.

### Safety

The majority of patients in all treatment arms experienced at least one AE (any grade) within 30 days that was deemed to be related to the study drug or WBRT. The most common (≥10 % of patients) treatment-emergent AEs, Grade 3/4 AEs (≥3 % of patients), and statistically significant AEs are listed in Table 3. Across all three arms the most common AEs were nausea (30, 22, 31 %), fatigue (22, 26, 21 %), alopecia (19, 15, 15 %), and headache (15, 18, 21 %). Pneumonia (6, 3, 2 %) and fatigue (4, 2, 2 %) were the most frequently reported Grade 3/4 AEs considered possibly related to study drug. There was a lower incidence for Grade 3/4 AEs in the veliparib arms (50 mg versus 200 mg; *p* < 0.05) compared with placebo. No new safety signals of veliparib were identified.

**Table 3:** Summary of adverse events

	Placebo + WBRT (*N* = 101)^a^	Veliparib 50 mg + WBRT (*N* = 103)	Veliparib 200 mg + WBRT (*N* = 102)
Any AE, n (%)	91 (90)	90 (87)	90 (98)
Any grade 3/4, n (%)	43 (43)	29 (28)^b^	26 (25)^b^
Any SAE, n (%)	39 (39)	31 (30)	36 (35)
Any fatal AE, n (%)	13 (13)	11 (11)	18 (18)
Any AE (≥10 % patients), n (%)			
Nausea	30 (30)	23 (22)	32 (31)
Fatigue	22 (22)	27 (26)	21 (21)
Alopecia	19 (19)	15 (15)	15 (15)
Headache	15 (15)	18 (17)	21 (21)
Decreased appetite	14 (14)	11 (11)	15 (15)
Dyspnea	14 (14)	8 (8)	11 (11)
Constipation	12 (12)	10 (10)	11 (11)
Insomnia	11 (11)	10 (10)	6 (6)
Asthenia	11 (11)	9 (9)	13 (13)
Dizziness	11 (11)	8 (8)	10 (10)
Malignant neoplasm progression	8 (8)	11 (11)	18 (18)
Any Grade 3/4 AE (≥3 % patients), n (%)			
Pneumonia	6 (8)	3 (3)	2 (2)
Fatigue	4 (4)	2 (2)	2 (2)
Pain	4 (4)	1 (1)	0 (0)
Anemia	3 (3)	1 (1)	2 (2)
Dehydration	3 (3)	0 (0)	1 (1)
Brain edema	3 (3)	0 (0)	0 (0)
Convulsion	3 (3)	0 (0)	0 (0)
Malignant neoplasm progression	2 (2)	2 (2)	4 (4)
Pulmonary embolism	1 (1)	4 (4)	2 (2)
Thrombocytopenia	1 (1)	3 (3)	2 (2)
Hyperglycemia	1 (1)	2 (2)	3 (3)
Statistically significant AEs, n (%)			
Vomiting	15 (15)	5 (5)^b^	11 (11)
Brain edema	6 (6)	1 (1)	0 (0)^b^
Dehydration	5 (5)	0 (0)^b^	1 (1)

*AE* adverse event, *SAE* serious adverse event, *WBRT* whole-brain radiation therapy

^a^Safety analysis population = 101

^b^ *p* < 0.05

## Discussion

This phase 2, randomized study evaluated the efficacy and safety of veliparib administered during WBRT for patients with brain metastases from NSCLC. Although preclinical and early clinical data suggested that veliparib might potentiate the efficacy of radiotherapy, no difference was observed in the assessed endpoints between WBRT combined with veliparib (50 or 200 mg) or placebo. Safety parameters demonstrated an AE profile similar to the placebo arm, with no new safety signals of veliparib identified. The phase 1 trial examining combination therapy with veliparib and WBRT in patients with brain metastases suggested that this therapy would be beneficial for this poor prognosis patient population. This finding was not validated within this phase 2 trial. Although the primary endpoint of the current study was not met, the results of the study may provide additional information that will prove valuable for the design of future trials involving patients with brain metastases from NSCLC.

In the reported trial, patients were deemed suitable candidates for WBRT based upon the judgement of the individual investigator. This decision took into account not only the number, size, and location of the metastases, but also the systemic tumor burden and other factors that would favor the use of WBRT over other therapeutic options (eg, SRS). From a global perspective, WBRT remains the most common standard of care for patients with this disease and is a part of contemporary guidelines. In addition, the role of WBRT continues to be evaluated in a number of clinical trials including the NRG (National Surgical Adjuvant Breast and Bowel Project, Radiation Therapy Oncology Group, and Gynecologic Oncology Group), which has two phase 3 trials (NRG-CC001 and NRG-CC003) evaluating the role of WBRT versus hippocampal avoidance (HA) WBRT [28]. These trials will test whether HA-WBRT decreases the risk for neurocognitive decline in patients undergoing WBRT and underscores the ongoing need to assess WBRT in the population of NSCLC patients with brain metastases.

Historically, randomized trials evaluating novel treatment options for patients with brain metastases have included a heterogeneous population of primary tumors [29, 30]. Typically, the mixture of primary tumors in these trials includes 50 % NSCLC, 15–20 % breast cancer, 15–20 % melanoma, and a few other tumor types [13]. The prognosis of patients and their response to local or systemic treatment differs amongst primary tumor types as well as histologic groups. The heterogeneity within trials, in addition to small patient numbers reported in historic trials, has complicated the identification of prognostic factors as well as the development of better treatments and methods to assess outcomes [29, 30].

In addition to heterogeneity in patient populations, responses have been inconsistently evaluated, making it challenging to compare findings across trials. The majority of trials have either used response criteria developed for solid tumors (e.g. RECIST [26, 27] or World Health Organization [31]) or modified versions of response criteria developed specifically for high-grade gliomas (Macdonald criteria [25], Response Assessment in Neuro-Oncology [RANO] [32]). Objective response assessment remains a common endpoint in phase 2 trials in patients with brain metastases given the likelihood for regulatory approval of the respective drug and or therapeutic regimen if response rates are favorable [33, 34].

The RANO working group recently developed recommendations to improve the design and implementation of clinical trials in patients with brain metastases [29, 30]. One of the consensus recommendations involved limiting the patient population of trials evaluating systemic treatments to a specific primary tumor type (or primary tumor subtype, if applicable). RANO also recommended that trials archive imaging studies with linked outcomes data to allow retrospective analyses comparing measurement techniques, response criteria, and associations with neurologic and survival outcomes [29].

This study was a randomized trial involving a relatively homogeneous population of patients with brain metastases from NSCLC. The majority of patients across all treatment groups had GPA scores ≤2.5 (88–90 %) and KPS > 80 (61–66 %). OS was not significantly different between WBRT plus either dose of veliparib (50 and 200 mg; 185 days each) or placebo (209 days). OS was also similar to estimated disease-specific GPA for patients with NSCLC and brain metastases [35]. The reported patient demographics and survival results are comparable with previously published data indicating the treated population was not atypical [23, 36]. Unfortunately, we did not have EGFR mutations and ALK rearrangements data for many patients, which greatly limited our ability to analyze the impact of EGFR and ALK on outcomes.

Recently a secondary analysis of a randomized trial comparing SRS alone to WBRT plus SRS in patients with brain metastases from NSCLC has been reported [37]. From a survival standpoint, patients with a favorable prognosis (eg, GPA score 2.5–4) had a significantly longer median survival with WBRT plus SRS (16.7 months) when compared to SRS alone (10.6 months; *p* = 0.04). This benefit was not observed in the unfavorable prognosis group (eg, GPA score <2.5; *p* = 0.86). Although there was not a statistical therapeutic advantage between WBRT plus either dose of veliparib or placebo in the reported trial, hazard ratio (HR) analyses in patients with GPA score >2.5 (HR 0.68, 95 % confidence interval [95 % CI] 0.24–1.91 and HR 0.65, 95 % CI 0.22–1.94) in comparison to patients with GPA score ≤ 2.5 (HR 1.03, 95 % CI 0.73–1.44 and HR 1.02, 95 % CI 0.73–1.36) with both 50 or 200 mg doses of veliparib, respectively is noteworthy (Fig. 2). However, this data should be interpreted with caution due to the limited number of patients with GPA score >2.5 (n = 11) in comparison to patients with GPA scores ≤2.5 (n = 91). Nevertheless, the benefit of WBRT observed in this study for patients with brain metastases from NSCLC (especially those with a favorable prognosis) warrants further investigation.

Improvement of OS in clinical trials for patients with brain metastases has been very difficult to achieve, except for patients with a single metastasis, as brain metastases may not be the main cause of death for these patients. Even if an OS endpoint might not be achievable in brain metastases studies, other endpoints evaluated in the current study suggest that there was no discernible effect of veliparib when used in combination with WBRT, including a lack of effect on the tumor response rate, radiographic brain metastasis progression, and time to clinical brain metastases progression; endpoints that do not depend on the status of systemic disease (i.e. disease that affects other parts of, or even the whole body). The number of deaths due to systemic disease progression of NSCLC was high, yet similar across all treatment arms (WBRT plus placebo BID, 49 %; WBRT plus 50 mg veliparib BID, 60 % and WBRT plus 200 mg veliparib BID, 56 %).

Further evaluation of the safety data showed that there was no mechanistic trend to support the improved Grade 3/4 events on the veliparib arms. This was likely due to a small number of events that by chance occurred more commonly on the placebo arm and/or happened to occur at Grade 2 as opposed to Grade 3/4, as the overall numbers of events were equivocal. There are no future plans to study veliparib in combination with WBRT in brain metastases, however ongoing clinical studies are evaluating veliparib in combination with different radiation and chemotherapy regimens in other tumor types and organs [38, 39].

Thus far, no medical therapies for patients with brain metastases have demonstrated a benefit in OS. Even with available treatment options (e.g. WBRT and stereotactic radiosurgery) prognosis remains very poor for the majority of patients. Novel therapies are needed to improve clinical outcomes for patients with brain metastases from NSCLC.

## Electronic supplementary material

Below is the link to the electronic supplementary material.

Supplementary material 1 (DOCX 92 KB)
