# Supplementary figures and images for: Veliparib in combination with whole-brain radiation therapy for patients with brain metastases from non-small cell lung cancer: results of a randomized, global, placebo-controlled study
Source: J Neurooncol. 2016 Sep 21;131(1):105–15. doi: 10.1007/s11060-016-2275-x (PMC5258788; doi:10.1007/s11060-016-2275-x)

**Supplemental data**

**Supplemental Table 1** Summary of exposure to study drug and WBRT


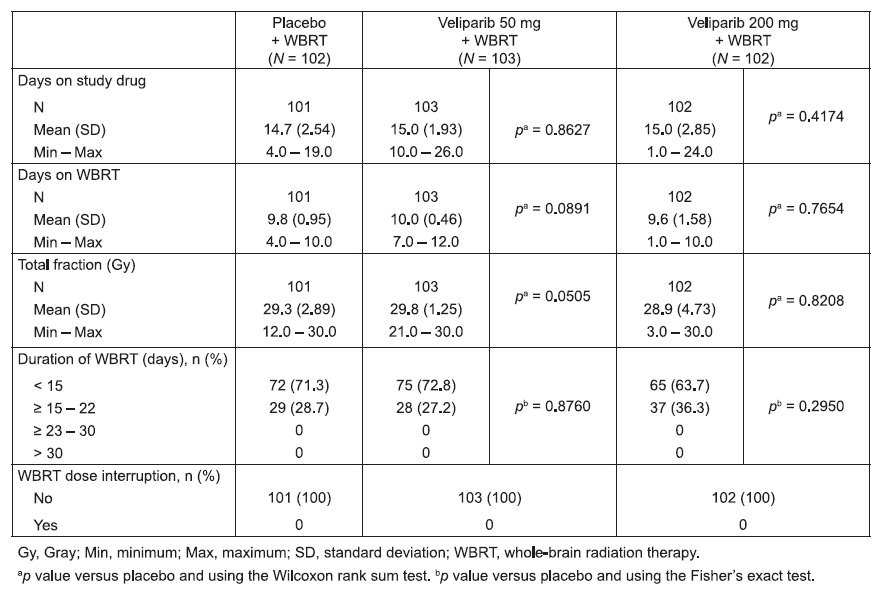

Supplement: Supplementary file 1 — Supplementary material 1 (DOCX 92 KB) [file 11060_2016_2275_MOESM1_ESM.docx]
